# Function of SREBP1 in the Milk Fat Synthesis of Dairy Cow Mammary Epithelial Cells

**DOI:** 10.3390/ijms150916998

**Published:** 2014-09-23

**Authors:** Nan Li, Feng Zhao, Chenjie Wei, Mengyao Liang, Na Zhang, Chunmei Wang, Qing-Zhang Li, Xue-Jun Gao

**Affiliations:** Key Laboratory of Agricultural Biological Functional Genes, Northeast Agricultural University, Harbin 150030, China; E-Mails: linan0829@163.com (N.L.); erjinzhi@126.com (F.Z.); chengjiewin@163.com (Che.W.); liangmengyao2008@sina.com (M.L.); nazhang1981@126.com (N.Z.); wangcm-1@sohu.com (Chu.W.); qzli@neau.edu.cn (Q.-Z.L.)

**Keywords:** SREBPs, mTOR, dairy cow mammary epithelial cells, milk fat synthesis

## Abstract

Sterol regulatory element-binding proteins (SREBPs) belong to a family of nuclear transcription factors. The question of which is the most important positive regulator in milk fat synthesis in dairy cow mammary epithelial cells (DCMECs) between SREBPs or other nuclear transcription factors, such as peroxisome proliferator-activated receptor γ (PPARγ), remains a controversial one. Recent studies have found that mTORC1 (the mammalian target of rapamycin C1) regulates SREBP1 to promote fat synthesis. Thus far, however, the interaction between the SREBP1 and mTOR (the mammalian target of rapamycin) pathways in the regulation of milk fat synthesis remains poorly understood. This study aimed to identify the function of SREBP1 in milk fat synthesis and to characterize the relationship between SREBP1 and mTOR in DCMECs. The effects of SREBP1 overexpression and gene silencing on milk fat synthesis and the effects of stearic acid and serum on SREBP1 expression in the upregulation of milk fat synthesis were investigated in DCMECs using immunostaining, Western blotting, real-time quantitative PCR, lipid droplet staining, and detection kits for triglyceride content. SREBP1 was found to be a positive regulator of milk fat synthesis and was shown to be regulated by stearic acid and serum. These findings indicate that SREBP1 is the key positive regulator in milk fat synthesis.

## 1. Introduction

Sterol regulatory element-binding proteins (SREBPs) belong to the basic helix-loop-helix-leucine zipper (bHLH-Zip) family of transcription factors, which are synthesized as inactive precursors bound to the endoplasmic reticulum (ER). SREBP precursors exit the ER and transit to the Golgi apparatus, where two distinct proteases cleave the SREBP precursor to release the transcriptionally active *N*-terminus. The *N*-terminus contains the bHLH-Zip region that, upon binding to DNA, functions as a transcription factor. Each SREBP precursor exists in a hairpin-like conformation of ~1150 amino acids (~125 kDa) organized into three domains: an NH_2_-terminal cytoplasmic domain that functions as a DNA-binding transcription factor; a central domain containing two transmembrane segments projecting into the lumen of the ER; and a COOH-terminal cytoplasmic domain that performs essential regulatory functions. The mammalian genome encodes three SREBP isoforms designated SREBP1a, SREBP1c, and SREBP2. SREBP1a and SREBP1c are encoded by the same gene (*SREBP1*), which is regulated by two distinct promoters and alternative splicing [1]. The original cloning of the *SREBP1* gene from human HeLa cells yielded several partial cDNA clones with alternative sequences at both the 5' and 3' ends, which were postulated to result from alternative splicing. *SREBP1a* and *SREBP1c* differ in sequence at both their 5' and 3' ends. *SREBP1a* consists of exons la, 18a, and 19a, while *SREBP1c* consists of exons lc, 18c, and 19c. Exons la and lc are separated by ~14 kb [2]. The two different sequences at each end were designated “a” and “c”. The full-length human cDNA containing “c” sequences at both ends was designated *SREBP1c* and the full-length cDNA isolated from Chinese hamster ovary (CHO) cells contained “a” sequences at both ends was designated *SREBP1a* [3]. The *N*-terminus contains a DNA activation domain which can bind transcriptional coactivators, such as CBP [4]. SREBP1 activates mainly genes encoding fatty acid (FA) and triglyceride biosynthesis: SREBP1a activates the genes required in the synthesis of cholesterol and fatty acid, such as fatty acid elongase and stearoyl-CoA desaturase, while SREBP1c directly activates the expression of more than 30 genes dedicated to the synthesis and uptake of fatty acids and triglycerides. The target genes of SREBP1c include low density lipoprotein (LDL) receptor, ACC (acetyl-CoA carboxylase), FAS (fatty acid synthase), SCD (stearoyl-CoA desaturase), INSIG1 (insulin induced gene-1), S14, GK (glucokinase), and PEPCK (phosphoenolpyruvate carboxykinase), which are involved in milk fat synthesis and glucose synthesis [5,6]. The target genes of SREBP2 include HMGCS (3-hydroxy-3-methylglutaryl-coenzyme a synthase), HMGCR (HMGC reductase), FDPS (farnesyl diphosphate synthase), SS (squalene synthase), and DNAJA4 (DNAJ (HSP40) homolog subfamily-A member-4) [7,8], which are involved in either the cholesterol biosynthesis pathway or in cholesterologenesis. When cells (such as liver cells) are stimulated by oxygen sterol, LXR (liver X receptor), NF-Y (nuclear factor-Y), and Spl (specificity protein 1) signal, SREBPs are activated. The inactive precursors are activated via a splicing system (SCAP, S1P, and S2P) consisting of a two-step photolytic cleavage by site-1 (S1P) and site-2 (S2P). Following cleavage, the amino-terminal fragments of SREBPs (nSREBP1c) translocate to the nucleus as homodimers and bind to sterol regulatory elements (SREs) within the promoters of target genes [9].

Ma and Corl found that SREBP1 plays an important role in the integrated regulation of lipid synthesis in dairy cow mammary epithelial cells (DCMECs) through regulation of key enzymes [10]. Milk fat is composed predominantly of triglycerides containing fatty acids that arise from two sources: *de novo* synthesis within the mammary gland and the uptake of long-chain fatty acids from circulation. SREBP1 regulates lipid synthesis in DCMECs by controlling the transcription of genes encoding enzymes involved in *de novo* FA synthesis, FA desaturation, long-chain FA uptake, and triglyceride esterification. Recent findings reported by Loor *et al*. [11] challenged the proposal that SREBP1 is central for milk fat synthesis regulation and highlight a pivotal role for a concerted action among *PPARG*, *PPARGC1A*, and *INSIG1*. Wan showed that the active compound in *Vaccaria segetalis* has similar functions as estrogen and/or prolactin in DCMECs, increasing the expressions of *prlr*, *erα*, *akt1*, and *elf5* genes, while repressing peroxisome proliferater-activated receptor (*PPARγ*) expression [12]. Findings reported by Huang indicate that SOCS3 acts as an inhibitor of the JAK2/STAT5a (janus kinase2/signal transducer and activator of transcription 5a) pathway and disturbs FA synthesis by decreasing SREBP1c expression, thus validating its involvement in fat synthesis [13]. Liu showed the liver expression of lipogenic genes including *SREBP1c* (560%), *FAS* (190%), *ACC* (48%), and *SCD1* (286%) to be elevated by ethanol. Luteolin was shown to reduce the ethanol-induced expression of these genes in the liver: SREBP1c (79%), FAS (80%), ACC (60%), and SCD1 (89%) in mice hepatocytes. Furthermore, the ethanol-induced reduction of AMP-activated protein kinase and SREBP1c phosphorylation was shown to be abrogated by luteolin [14]. From the above results it is clear that there is still controversy regarding the question of which is the most important positive regulator in milk fat synthesis in DCMECs between SREBPs or other nuclear transcription factors, such as PPARγ.

A role for mTOR, the mammalian target of rapamycin, in promoting protein synthesis has been well described, and SREBP is known to play an important role in regulating lipid synthesis [15]. Studies have shown that insulin drives hepatic lipogenesis by inducing SREBP1c, and the inhibition of mTORC1 by rapamycin has been shown to dramatically reduce the expression of SREBP1c *in vitro* and *in vivo*, indicating that mTORC1 plays an important role in controlling SREBP1c expression [16,17,18]. Previous work has also shown that mTORC1 positively regulates the activity of SREBP1 and that the mTOR pathway regulates several anabolic and catabolic pathways at the mRNA expression level [18,19,20]. However, rapamycin does not affect SREBP target gene expression in all cellular contexts [15,21]. It was recently demonstrated that mTORC1 regulates SREBP by controlling the nuclear entry of lipin 1, a phosphatidic acid phosphatase [22]. Although there are some reports about the role of SREBP1c in mouse and human, the molecular events associated with the regulation of milk fat synthesis in DCMECs remain largely unknown. The aim of this study was to investigate the functional role of SREBP1 in milk fat synthesis in DCMECs and to determine whether SREBP1 interacts with the mTOR pathway to regulate milk fat synthesis.

## 2. Results and Discussion

### 2.1. Sterol Regulatory Element-Binding Protein 1 (SREBP1) Overexpression in Dairy Cow Mammary Epithelial Cells (DCMECs) Increases the Expression of Lipogenic Genes and Key Enzymes of Fatty Acid Synthesis as Well as Triglyceride Secretion

**Figure 1:**
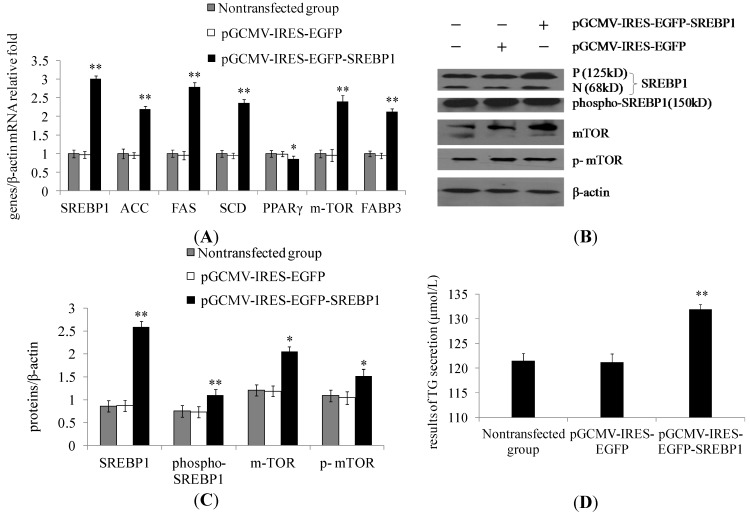
Effect of sterol regulatory element-binding protein 1 (SREBP1) overexpression on milk fat synthesis in dairy cow mammary epithelial cells (DCMECs). Three groups of DCMECs were assessed: nontransfected group, pGCMV–IRES–EGFP empty vector group (control group) and pGCMV–IRES–EGFP–SREBP1 group. (**A**) Relative mRNA levels (gene of interest/β-actin) of indicated genes were determined by qRT-PCR after gene overexpression of *SREBP1*; (**B**) Expression levels of indicated proteins were assessed by Western blotting. P and N denote the precursor and cleaved nuclear forms of SREBP1, respectively; (**C**) Quantification of protein of interest/β-actin relative fold by gray scale scan; and (**D**) Triglyceride (TG) content in the culture supernatant of DCMECs. Values represent means ± SE (standard error) (*n* = 3). * p < 0.05, ** p < 0.01 compared with the pGCMV–IRES–EGFP empty vector group.

The mRNA expression of *SREBP1*, *ACC*, *FAS*, *SCD*, *m-TOR*, and *FABP3* (fatty acid-binding protein) was significantly increased in the pGCMV–IRES–EGFP–SREBP1 group compared with the empty vector group (Figure 1A), whereas *PPARγ* was found to be downregulated. The protein expression levels of SREBP1, p-SREBP1, mTOR, and p-mTOR were notably increased in cells transfected with SREBP1 compared with cells in the empty vector group (Figure 1B,C). Overexpression of SREBP1 in DCMECs was found to significantly increase triglyceride secretion (Figure 1D). These findings reveal that the overexpression of SREBP1 increases milk fat synthesis in DCMECs.

### 2.2. SREBP1 Gene Silencing Decreases the Expression of Lipogenic Genes and Key Enzymes of Fatty Acid Synthesis and Decreases Triglyceride Secretion in DCMECs

**Figure 2:**
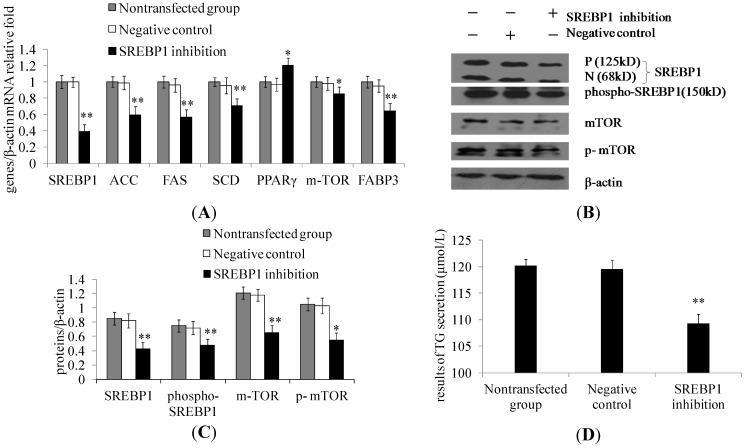
Effect of *SREBP1* gene silencing on milk fat synthesis in DCMECs. Three groups of DCMECs were assessed: nontransfected group, negative group (control group), SREBP1 siRNA-transfected group. (**A**) Relative mRNA levels (gene of interest/β-actin) of indicated genes were determined by qRT-PCR after gene silencing of *SREBP1*; (**B**) Expression levels of indicated proteins were assessed by Western blotting. P and N denote the precursor and cleaved nuclear forms of SREBP1, respectively; (**C**) Quantification of protein of interest/β-actin relative fold by gray scale scan; and (**D**) TG content in the culture supernatant of DCMECs. Values represent means ± SE (*n* = 3). * p < 0.05, ** p < 0.01 compared with negative control group.

SREBP1 knockdown by siRNA transfection was verified using qRT-PCR. *SREBP1* gene silencing decreased the mRNA expression levels of *SREBP1*, *ACC*, *FAS*, *SCD*, *mTOR*, and *FABP3* in cells transfected with *SREBP1* siRNA compared with cells transfected with the empty vector, while *PPARγ* was up-regulated in *SREBP1* siRNA-transfected cells (Figure 2A). The protein expression levels of SREBP1, p-SREBP1, mTOR, and p-mTOR were notably decreased in cells transfected with *SREBP1* siRNA compared with cells in the negative control group (Figure 2B,C). *SREBP1* gene silencing in DCMECs was further found to result in significantly decreased triglyceride secretion (Figure 2D). These findings reveal that gene silencing of *SREBP1* decreases milk fat synthesis in DCMECs.

**Figure 3:**
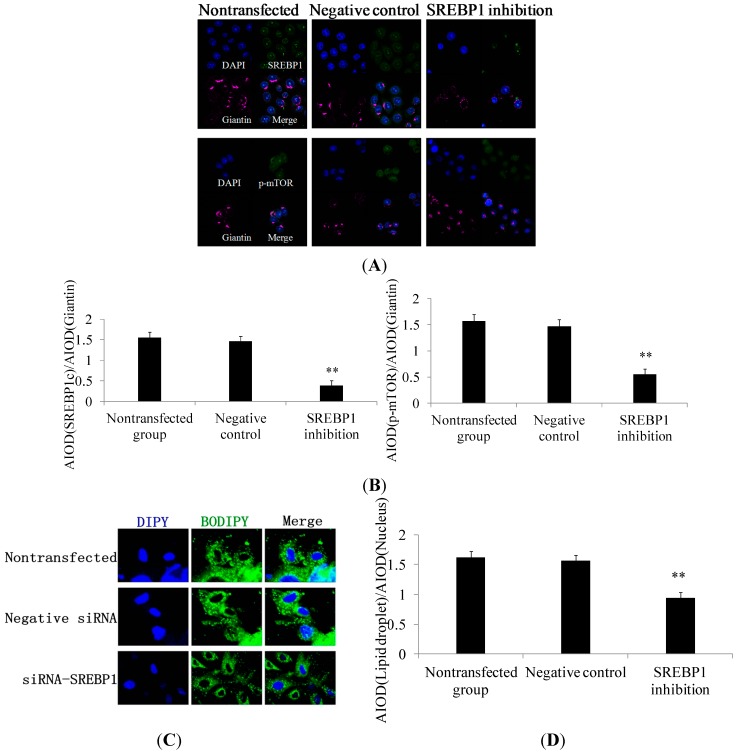
Effect of SREBP1 gene silencing on the subcellular localization of molecules related to milk fat synthesis in DCMECs. Groups of DCMECs were assessed as in Figure 2. (**A**) Localization and integral optical density analysis of SREBP1 and p-mTOR. SREBP1 and p-mTOR (green), giantin (purple), and DAPI (blue). Scale bars = 10 μm; (**B**) Integral optical density analysis of SREBP1 and p-mTOR. p-mTOR (green), giantin (purple), and DAPI (blue). Scale bars = 10 μm; and (**C**,**D**) Immunofluorescent staining and integral optical density analysis of lipid droplets were performed on DCMECs. Lipid droplet (green) and DAPI (blue). Scale bars = 10 or 15 μm. Values represent means ± SE (*n* = 3). ** *p* < 0.01 compared with negative control group.

Lower expression levels of SREBP1 (Figure 3A,B) and p-mTOR (Figure 3C,D) in response to *SREBP1* gene silencing were also demonstrated by immunofluorescence microscopy. *SREBP1* gene silencing in DCMECs was further found to result in significantly decreased lipid droplet content (Figure 3E). These results are in accord with the aforementioned conclusion, further revealing SREBP1 as a key positive regulator in milk fat synthesis in DCMECs.

### 2.3. The Effect of Stearic Acid and Serum on SREBP1 and mTOR Expression in the Regulation of Milk Fat Synthesis

**Figure 4:**
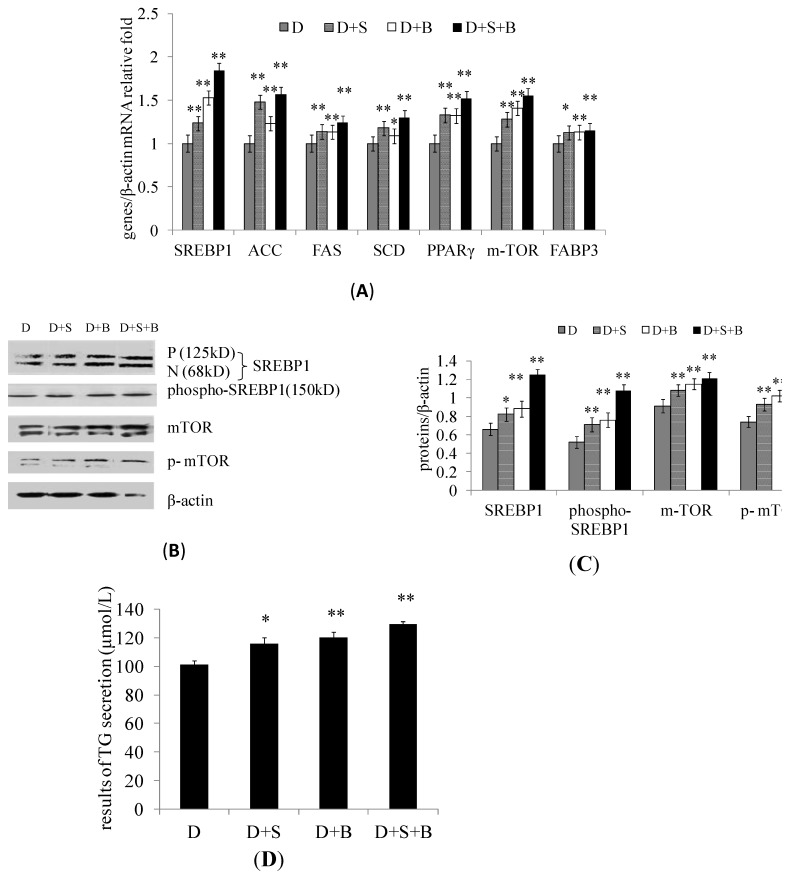
The effect of stearic acid and serum on the regulation of milk fat synthesis by SREBP1. Four groups of DCMECs were assessed: Untreated cells grown in DF12 media (control group, D), DCMECs treated with stearic acid (0.1 mM) + DF12 (D + S), DCMECs treated with DF12 and 10% FBS (D + B), and DCMECs treated with stearic acid (0.1 mM) + DF12 + 10% FBS (D + S + B). In each group cells were treated for 24 h. (**A**) Relative mRNA levels (gene of interest/β-actin) of the indicated genes were determined by qRT-PCR; (**B**) The expression levels of the indicated proteins were assessed by Western blotting. P and N denote the precursor and cleaved nuclear forms of SREBP1, respectively; (**C**) Quantification of protein of interest/β-actin relative fold by gray scale scan; and (**D**) TG content in the culture supernatant of DCMECs. Values represent means ± SE (*n* = 3). * p < 0.05, ** p < 0.01 compared with negative control group.

To determine the effect of stearic acid and serum on lipogenic gene expression in DCMECs, cells were treated with stearic acid (100 μmol·L^−1^) and serum (10% FBS), and were harvested at 24 h after treatment. The mRNA levels of *SREBP1*, *ACC*, *FAS*, *SCD*, *PPARγ*, *mTOR*, and *FABP3* were found to be up-regulated in cells treated with stearic acid (D + S), in cells treated with FBS (D + B), and in cells treated with stearic acid + FBS (D + S + B) compared with untreated (D) cells (Figure 4A). As shown by Western blotting analysis, the protein levels of SREBP1, p-SREBP1, mTOR, and p-mTOR were all increased in the D + S, D + B, and D + S + B groups compared with the D group (Figure 4B,C). Stearic acid treatment resulted in obvious notable increase in triglyceride secretion (Figure 4D) in DCMECs. These findings indicate that stearic acid and serum activate SREBP1 to enhance milk fat synthesis, and that the mTOR signaling pathway is involved in the observed effect.

**Figure 5:**
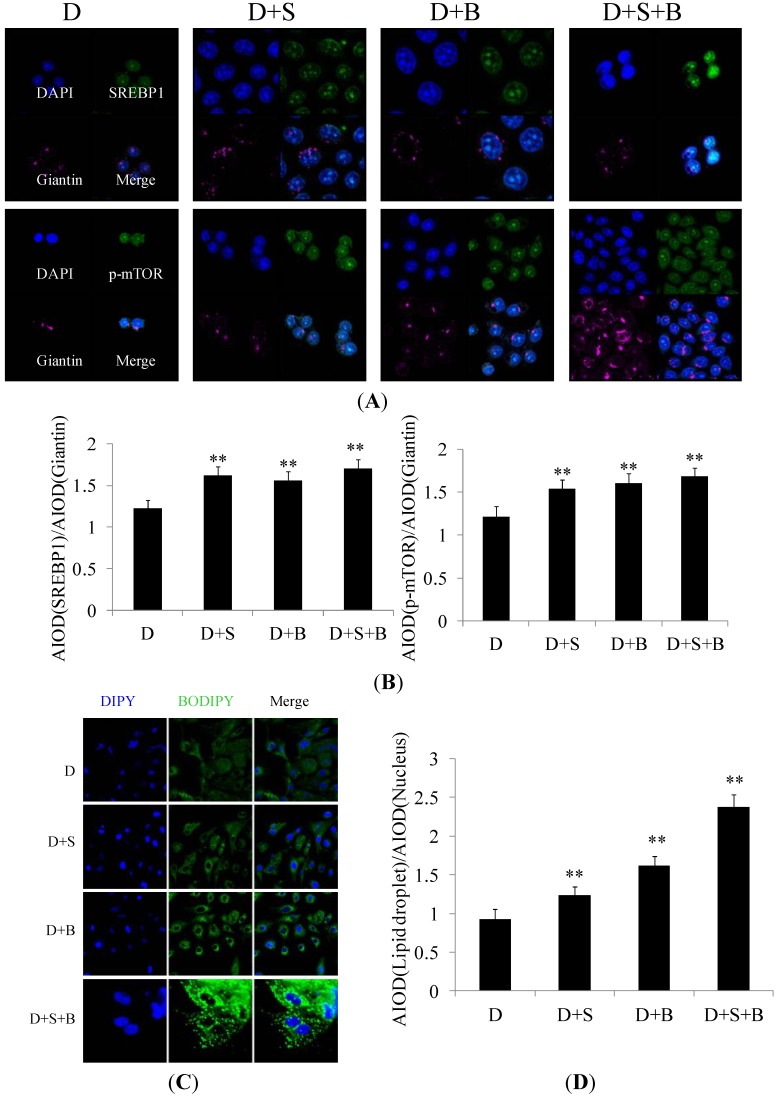
The effect of stearic acid and serum on the subcellular localization of molecules related to milk fat synthesis. Groups of DCMECs were assessed as in Figure 4. (**A**) Localization of SREBP1 and p-mTOR. SREBP1 (green), giantin (purple), and DAPI (dark blue). Scale bars = 10 μm; (**B**) Integral optical density analysis of p-mTOR. p-mTOR (green), giantin (purple), and DAPI (blue). Scale bars = 10 μm; and (**C**,**D**) Immunofluorescent staining and integral optical density analysis of lipid droplets. Lipid droplet (green) and DAPI (blue). Scale bars = 10, 15, 45 or 60 μm. Values represent means ± SE (*n* = 3). ** p < 0.01 compared with negative control group.

In agreement with the Western blotting results, the expression levels of SREBP1 and p-mTOR were also shown by immunofluorescence microscopy to be increased in response to stearic acid treatment (Figure 5A,B). Stearic acid treatment resulted in obvious notable increase in lipid droplet formation (Figure 5C,D) in DCMECs. These results are in accord with the aforementioned conclusion, further revealing the activities of SREBP1 and mTOR sense to stearic acid and serum to enhance milk fat synthesis.

### 2.4. Discussion

The SREBP family of transcription factors is critical to the regulation of fatty acid and cholesterol biosynthetic gene expression [23]. Studies by Brown, Goldstein, and colleagues have elucidated key parts of the mechanism by which cholesterol sensing is coupled to the activity of SREBPs [24]. Few reports on phospho-SREBP1 (p-SREBP1) exist. It has been shown that SREBPs are inactive precursors and that the amino-terminal fragment of SREBP (nSREBP1c) is the active form of the protein and translocates to the nucleus. A newly generated p-SREBP1c-specific Ser372 antibody was used in this study. Li demonstrated that Ser372 phosphorylation of SREBP1c by AMPK (AMP-activated protein kinase) is necessary for inhibition of the proteolytic processing and transcriptional activity of SREBP1c. The phosphorylation of SREBP1c is an inactivation mechanism. AMPK stimulates Ser372 phosphorylation, suppresses SREBP1c cleavage and nuclear translocation, and represses SREBP1c target gene expression in hepatocytes exposed to high glucose, leading to reduced lipogenesis and lipid accumulation [25]. In this study, phosphorylated SREBP1 was found to be associated with increasing or decreasing SREBP1 expression. This may be interpreted as a dynamic balance between the nonphosphorylated, active form and the phosphorylated, inactive form.

Using *SREBP1* overexpression and gene silencing experiments we have demonstrated that SREBP1 increases the relative mRNA level of *FABP3.* Fatty acid binding protein (FABP) and acyl-CoA binding protein (ACBP) are the main intracellular FA transporters in nonruminant cells [26]. The former has a high affinity for long-chain FA (LCFA) but also binds acyl-CoA [27,28]. In the current study, we confirm that SREBP1 regulates the expression of FABP3 to affect milk fat synthesis.

SREBP1 over-expression resulted in an increase in the mRNA levels of *ACC*, *FAS*, *SCD*, *m-TOR*, and *FABP3* and a slight decrease in *PPARγ* mRNA, while *SREBP1* gene silencing had the opposite effect. PPARγ is a member of the PPAR family of transcription factors and nuclear receptors and plays a pivotal part in cell fate determination, lipid biosynthesis, inflammation, and insulin sensitivity [29]. Recently reported findings suggest that the activation of PPARγ and PPARγ reduces triacylglycerol synthesis in rat hepatoma cells by reducing nuclear SREBP1 levels [30]. Our results further indicate that SREBP1 may be a negative regulator of PPARγ in DCMECs, and we speculate that PPARγ exercises its role in balancing milk fat synthesis.

Lipid synthesis involves the *de novo* synthesis of FA as well as the incorporation of *de novo* and preformed FA into triglycerides. The triglycerides accumulate to form lipid droplets, primarily in the liver, adipose, and mammary gland tissues of mammals. The activation of these metabolic pathways requires the coordinated regulation of a network of genes encoding lipogenic enzymes, such as the *de novo* FA synthesis genes *FAS* and *ACC*, as well as the FA modification gene *SCD*. SREBP1 translocates to the nucleus where it activates lipogenic genes by binding to the SREBP1 response element of target genes, such as ACC and FAS [31]. The mRNA expression levels of *ACC*, *FAS* and *SCD*, as well as triglyceride secretion and lipid droplets represent the cells’ capacity for lipid synthesis. It was previously noted that SREBP1 activates ACC, FAS, and SCD [5], and in this study, we observed that SREBP1 affects the mRNA expression levels of the *ACC*, *FAS*, and *SCD* genes in accordance with changes in triglyceride content and lipid droplet accumulation, further confirming that SREBP1 acts on its target genes to regulate milk fat synthesis in DCMECs.

In cells in which SREBP1 was over-expressed, the protein levels of m-TOR, and p-mTOR were elevated, whereas the opposite effects were observed when the *SREBP1* gene was silenced. Immunofluorescence was used to assess the localization of SREBP1 and p-mTOR, and revealed that *SREBP1* gene silencing reduces the activity of p-mTOR protein in DCMECs. Our results suggest that the expression and subcellular localization of p-mTOR are regulated by SREBP1. Considering that p-mTOR regulates SREBP1 activity, our findings also suggest that a positive feedback loop may exist to regulate the SREBP1 and mTOR signaling pathways in DCMECs: SREBP1 over-expression promotes mTOR signaling in milk fat synthesis of DCMECs, whereas gene silencing of *SREBP1* weakens the mTOR signaling pathway.

LCFAs have been identified as an important cellular source of metabolic energy, as a substrate for membrane biogenesis (phospholipid), and for storage of metabolic energy (triglycerides and cholesterol esters) [26]. Serum and glucose or amino acid deprivation are known to strongly down-regulate SREBP target gene expression compared with cells grown in complete media or media lacking only serum [22]. We, thus, assessed the effect of stearic acid and serum on milk fat synthesis in DCMECs to ascertain whether SREBP1 is associated with the regulation of milk fat synthesis. Our experiments revealed that *ACC*, *FAS*, *SCD*, *PPARγ*, *m-TOR*, and *FABP3* mRNA levels were significantly increased by the addition of stearic acid and serum. Several LCFAs are natural ligands of non-ruminant PPARγ, which, along with its lipogenic target genes, is up-regulated in bovine mammary tissue during lactation [32]. Unlike serum deprivation alone, serum deprivation combined with stearic acid deprivation was shown to down-regulate these genes. The protein levels of SREBP1, p-SREBP1, m-TOR, and p-mTOR were shown to increase after the addition of stearic acid and serum, as were the triglyceride secretion and lipid droplet formation. From these findings, we conclude that fatty acids such as stearic acid and components in serum including lipogenic hormones activate the SREBP1 and mTOR signaling pathways to enhance milk fat synthesis in DCMECs.

## 3. Experimental Section

### 3.1. Experimental Materials

Instruments: Confocal microscope (TCS-SP2 AOBS, Leica, Wetzlar, Germany); ABI PRISM 7300 RT-PCR System (Applied Biosystems, Foster City, CA, USA).

Reagents: BODIPY 493/503 (Invitrogen, Carlsbad, CA, USA), Trizol (Invitrogen); Dulbecco’s Modified Eagle medium-F12 (DF-12, Gibco, Carlsbad, CA, USA); fetal bovine serum (FBS, Gibco); insulin, prolactin, hydrocortisone and stearic acid (Sigma, MO, USA); nitrocellulose membrane (Bio-RAD, Shanghai, China); BCA Protein Quantitative Detection kit (GENMED Co., Ltd., Shanghai, China); monoclonal antibody for phospho-SREBP1, mTOR, p-mTOR (Cell Signaling Technology, Beverly, MA, USA); monoclonal antibody for SREBP1, giantin and β-actin (Santa Cruz Biotechnology Inc., Santa Cruz, CA, USA); HRP-conjugated anti-rabbit IgG, HRP-conjugated anti-goat IgG, HRP-conjugated anti-mouse IgG (ZSGB-BIO, Beijing, China); Super ECL Plus Detection Reagent (ApplyGEN, Beijing, China); triglyceride GPO-POD assay kit (ApplyGEN, Beijing, China).

### 3.2. Primary DCMEC Culture and Treatment

Primary DCMECs were cultured and purified according to previously reported methods [33,34]. Purified DCMECs were cultured in DF-12 media containing 10% FBS, insulin (bovine, 5 μg·mL^−1^), hydrocortisone (1 μg·mL^−1^), prolactin (bovine, 5 μg·mL^−1^), penicillin (100 U·mL^−1^), and streptomycin (0.1 mg·mL^−1^). Before DCMEC treatment, culture medium was replaced with DF-12 media containing insulin, hydrocortisone, and prolactin (concentrations as above). For experimental assays, DCMECs in the logarithmic growth phase were plated at 3 × 10^4^ cells·cm^−2^.

### 3.3. Gene Silencing of SREBP1

DCMECs were transfected with SREBP1 siRNA or a negative scrambled control (GenePharma Co., Ltd., Shanghai, China) using Lipofectamine TM 2000 (LF2000, Invitrogen) according to the manufacturer’s instructions. We screened siRNA-SREBP1a (sense5'-GAGGCCAAGUUGAAUAAAUTT-3'; antisense5'-AUUUAUUCAACUUGGCCUCTT-3'), siRNA-SREBP1b (sense5'-GCUCCUCACUUGAAGGCUUTT-3'; antisense5'-AAGCCUUCAAGUGAGGAGCTT-3'), siRNA-SREBP1c (sense5'-GGAGGGUAUUCCUACAUGATT-3'; antisense5'-UCAUGUAGGAAUACCCUCCTT-3') and selected siRNA-SREBP1a for the highest gene silencing efficiency. The negative scrambled control siRNA (sense5'-UUCUCCGAACGUGUCACGUTT-3'; antisense5'-ACGUGACACGUUCGGAGAATT-3') had no significant homology to any gene of DCMECs. For silencing of *SREBP1* gene expression, 1 µg of siRNA and 2 µL of LF2000 were diluted in 200 µL of OPTI-MEMI medium, and added to the wells in 6-well plates to be transfected. Transfected cells were cultivated for 24 h, after which they were collected for further experiments.

### 3.4. pGCMV–IRES–EGFP–SREBP1 Construction and Transfection

Total RNA from cultured DCMECs was extracted using Trizol reagent and cDNA was synthesized. Bos taurus sterol regulatory element binding transcription factor 1 (SREBF1) mRNA (NM_001113302.1) is 3983 bp in length and encodes 1146 amino acids, with the CDS (coding sequence ) region extending from 50 to 3490 bp. The S2P cleavage site on the human and bovine SREBP1 protein is accompanied by the amino acid recognition sequence DRSRLALC. The cleavage site is situated between the amino acids L and C [29]. In this study, the sequence base pairs 50 to 1467 of bovine *SREBP1* mRNA were therefore amplified. This sequence encodes the *N*-terminus of the protein, a transmembrane domain, which enters the nucleus and binds to SREs (sterol regulatory elements) within the promoters of target genes.

The resulting PCR product was inserted into the pMD18-T plasmid (TaKaRa, Dalian, China), after which the plasmid identity was confirmed by digestion with the restriction enzymes *Nhe*I and *Hind*III (TaKaRa, Dalian, China) and by DNA sequencing. The *SREBP1* gene was cloned into the pGCMV–IRES–EGFP vector (GenePharma Co., Ltd., Shanghai, China). The recombinant plasmids were assessed by digestion with *Nhe*I and *Hind*III. Primers were designed with particular restriction enzyme sites to allow for the complete coding region of SREBP1 to be cloned. Forward primer: 5'-CTAGCTAGCATGGACGAGCCACCCTTCAAC-3' (*Nhe*I); Reverse primer: 5'-CCCAAGCTTAGCGGCTCTGGATTCACCTG-3' (*Hind*III). For optimal amplification, annealing and extension steps were carried out at 55.7 and 72 °C, respectively, for 35 cycles.

Transient transfections were carried out as previously described by Lu [35]. Briefly, DCMECs were transfected with pGCMV–IRES–EGFP–SREBP1 or with an empty vector using Lipofectamine TM 2000 (LF2000, Invitrogen, Camarillo, CA, USA) according to the manufacturer’s instructions. Nontransfected cells were used as controls. Cells were cultivated for 24 h, after which they were collected for further experiments.

### 3.5. BODIPY Staining of Lipid Droplets

Cells were washed with PBS, fixed with 3% formaldehyde for 15 min, and stained with BODIPY 493/503 (Invitrogen, stock concentration 1 mg/mL, working solution 1:1000 dilution) for 15 min at room temperature. Cells were then mounted with Prolong gold anti-fade reagent (Invitrogen) followed by three washes in phosphate buffered saline (PBS) [36].

### 3.6. Detection of Triglyceride Secretion

Cell-free supernatants were employed in triglyceride quantification using the TG GPO-POD Assay kit (Applygen Tech Inc., Beijing, China) according to the manufacturer’s instructions.

### 3.7. RNA Extraction and Real-Time Quantitative PCR

Total RNA from DCMECs was extracted using Trizol reagent according to the manufacturer’s instructions. Total RNA (1 μg) was transcribed into cDNA using Thermoscript Reverse Transcriptase (TaKaRa, Dalian, China) according to the manufacturer’s instructions. After reverse transcription, PCR reactions were carried out using an ABI PRISM 7300 RT-PCR System. β-actin was used as a reference gene. The primers of these gene transcripts were as follows: SREBP1: sense5'-ACGCCATCGAGAAACGCTAC-3', antisense5'-GTGCGCAGACTCAGGTTCTC-3'; ACC: sense5'-GGGTGAAAGACTGGGTTGAA-3', antisense5'-GACAGAGCACGGATGTGATG-3'; FAS: sense5'-CTGCAACTCAACGGGAACTT-3', antisense5'-AGGCTGGTCATGTTCTCCAG-3'; SCD: sense5'-CCCTTTCCTTGAGCTGTCTG-3', antisense5'-ATGCTGACTCTCTCCCCTGA-3'; PPARγ: sense5'-TCAAAGTGGAGCCTGTATC-3', antisense5'-CATAGTGGAACCCTGACG-3'; FABP3: sense5'-GAACTCGACTCCCAGCTTGAA-3', antisense5'-AAGCCTACCACAATCATCGAAG-3'; mTOR: sense5'-ATGCTGTCCCTGGTCCTTATG-3', antisense5'-GGGTCAGAGAGTGGCCTTCAA-3'; β-actin: sense5'-AAGGACCTCTACGCCAACACG-3', antisense5'-TTTGCGGTGGACGATGGAG-3'. RT-PCR analysis was performed using the ∆∆*C*_t_ method [37].

### 3.8. Immunofluorescence Assay

Immunofluorescence assays were performed using standard techniques [13] to assess SREBP1 and p-mTOR localization.

### 3.9. Western Blotting Analysis

Western blotting analysis was performed using standard techniques [12] to detect the expression of SREBP1, p-SREBP1, mTOR, p-mTOR, and β-actin.

### 3.10. Bioinformatics Analysis

The mRNA sequence of Bos SREBP1 was sourced from the NCBI nucleotide database (http://www.ncbi.nlm.nih.gov/nuccore) and the SMART online tool (http://smart.embl-heidelberg.de) was used to analyze the structure and function of the SREBP1 protein.

### 3.11. Statistical Analysis

Results were reported as mean ± SE. Data statistics and individual differences among groups were analyzed by using IBM SPSS Statistics 21 software (IBM, Armonk, New York, NY, USA). A statistical comparison of the means among the groups was performed using one-way analysis of variance. Differences between the means of individual groups were analyzed by Tukey *post hoc* tests. Differences with *p* < 0.05 were considered statistically significant and those with *p* < 0.01 were considered extremely significant. The integral optical densities of laser confocal images were analyzed using Image-Pro Plus 6.0 software (Media Cybernetics, Inc., Rockville, MD, USA) and the gray-scale scanning of Western blots was analyzed using Glyko Band Scan 5.0 software (Glyko, Hayward, CA, USA).

## 4. Conclusions

Together, these results reveal that SREBP1 is a positive regulator to promote milk fat synthesis in DCMECs. These data also suggest that there is a positive feedback-loop regulation between SREBP1 and mTOR signaling pathways in DCMECs. SREBP1 promotes the mTOR signaling pathway and milk fat synthesis of DCMECs. In the other hand, stearic acid and serum can up-regulate SREBP1 to enhance milk fat synthesis.
